# Simulation of the Performance of a Fundamental Neutron Physics Beamline at the High Flux Isotope Reactor

**DOI:** 10.6028/jres.110.017

**Published:** 2005-06-01

**Authors:** Rob Mahurin, Geoffrey Greene, Paul Kohler, Vince Cianciolo

**Affiliations:** University of Tennessee, Knoxville, TN 37996, USA; University of Tennessee, Knoxville, TN 37996, USA; Oak Ridge National Laboratory, Oak Ridge, TN 37831, USA; Oak Ridge National Laboratory, Oak Ridge, TN 37831, USA

**Keywords:** neutron optics, beamline simulation

## Abstract

We study the expected performance of the proposed fundamental neutron physics beamline at the upgraded High Flux Isotope Reactor at Oak Ridge National Laboratory. A curved neutron guide transmits the neutrons from the new cold source into a guide hall. A novel feature of the proposed guide is the use of vertical focussing to increase the flux for experiments that require relatively small cross-section beams. We use the simulation code IB to model straight, multi-channel curved, and tapered guides of various m values. Guide performance for the current NPDGamma and proposed abBA experiments is evaluated.

## 1. Neutron Physics at HFIR

The High Flux Isotope Reactor [1] at Oak Ridge National Laboratory is an 85 MW “flux-trap” reactor designed for the production of transuranium isotopes. The reactor first came on-line in 1966, underwent an upgrade in the late 1980s, and is presently undergoing a second refurbishment; during running periods the reactor is typically active for ≈ 5000 hours a year. When this upgrade is complete HFIR will be comparable to the most intense continuous source of cold neutrons in the world. The HFIR refurbishment includes the construction of a new guide hall, which will contain the fundamental physics beamline modelled here.

In this paper we evaluate the simulated guides based on their expected performance for the abBA [2] and NPDGamma [3] experiments. The figure of merit used for the abBA experiment is the neutron capture flux (n/cm^3^) in a small volume located ≈2.5 m from the end of the guide. For the NPDGamma experiment the figure of merit is the neutron fluence (n/s) in a circular aperture ≈10 cm in diameter located 2 m from the end of the guide.

The reader should note that these simulations only deal with the behavior of the guide, and do not account for losses due to windows and other effects.

## 2. The Cold Source and Upstream Guide

**Fig. 1:**
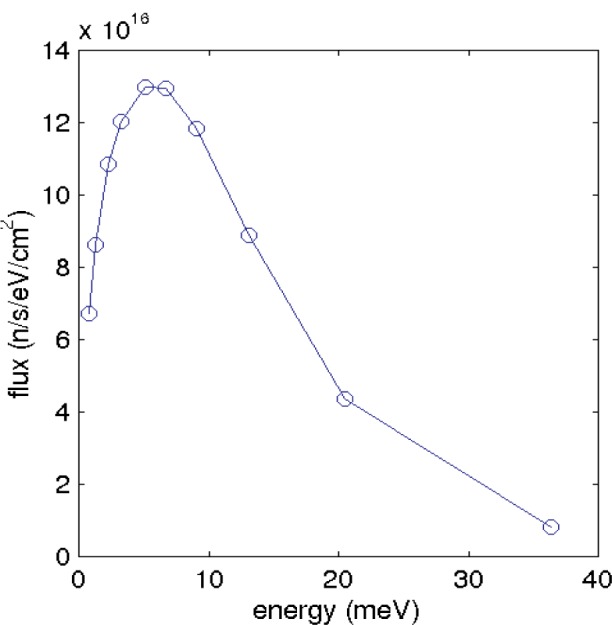
The spectrum of the cold moderator [4]. The integrated flux is 2.2 × 10^15^ n/s/cm^2^.

The cold moderator at HFIR is a square 8.52 cm on a side whose spectrum is shown in Fig. 1. The CG4 guide, which begins 5.297 m from the cold source, is a 15 m long, 1.9 cm wide, 4 channel bender with radius 107.43 m and supermirror parameter *m* = 2.5, followed by a 10 m long, *m* = 2 straight section. The bender tapers from 11.56 cm to 15 cm tall over the first 6 m of its length. At this point there is a gap for a triple-axis spectrometer, followed by about 10 m of guide leading to the fundamental physics experiments. Only the guide parameters of the section after this gap were varied.

The design calculations of the cold source and the CG4 guide [4] predict a value for the flux at 30 m of 1.97 × 10^9^ n/s/cm^2^. The simulations described here give a flux 1.90 × 10^9^ n/s/cm^2^, an agreement within about 5 %.

## 3. The Fundamental Neutron Physics Beamline

Extending the guide poses two challenges. One is space: the CG4 guide is nearly parallel to the adjacent beamline, and a straight extension would not leave adequate room for experiments. This problem is addressed by having a bender in the extension. The second problem is that the CG4 guide has an unfavorable aspect ratio (1.9 × 15 cm^2^) for the proposed fundamental physics experiments, which require fairly small cross-section beams. This problem is addressed by the vertical focussing.

The following parameters were varied to maximize the figure-of-merit “signals” described above:

- the entrance size
- the clearance from the adjacent beam line
- the channel width
- the vertical taper.

These degrees of freedom are largely independent of each other. The results of these simulations are described here.

### 3.1 Entrance Size

**Fig. 2:**
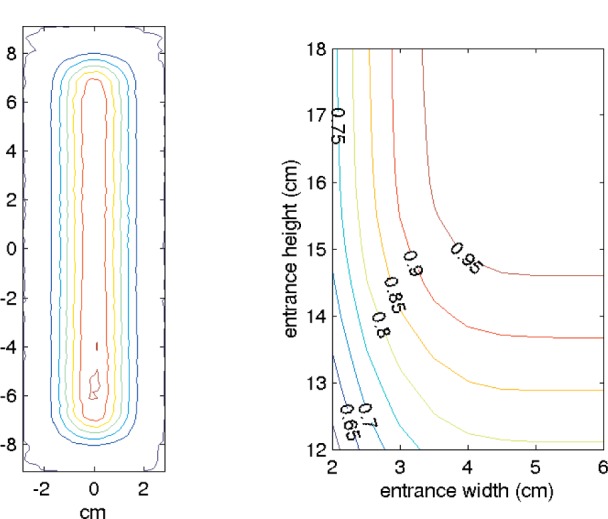
(a) Beam profile at the entrance to the fundamental physics beamline, ≈31.8 m from the moderator. The increased intensity at the bottom of the beam is due to the neutrons’ gravitational fall. (b) Curves in this panel are labeled with the fraction of incident neutrons accepted for the given horizontal and vertical aperture.

Figure 2 shows the neutron intensity at the entrance to the fundamental physics beamline, and the fraction of the incident beam that enters a different-sized guides. We chose to model guides with 4 × 15 cm^2^ entrances, keeping 95 % of the available neutrons.

### 3.2 Clearance From the Adjacent Guide

A clearance of ≈1.1 m from the undeflected guide path was desirable to have adequate space for the experiments. In this paper we consider only guides that accomplish this using a single bender. Table 1 summarizes the effect of bending the beam to attain this deflection. The guides in this table have the optimum number of channels, described in the next section.

### 3.3 Optimizing Channel Width

A single-channel bender is characterized by four parameters: the channel width *d*, the radius of curvature *r*, the length *L*, and the supermirror parameter *m*. It is convenient to define a sight angle $\gamma =\sqrt{2d/r}$, which allows one to consider the guides in a scale-free way [5]. For a 4 cm wide *m* = 3.5 bender illuminated by the HFIR spectrum, the optimum simulated *γ* ≃ 9.5 mrad. The transmission is then roughly the reflectivity raised to the number of bounces along the guide.

**Fig. 3:**
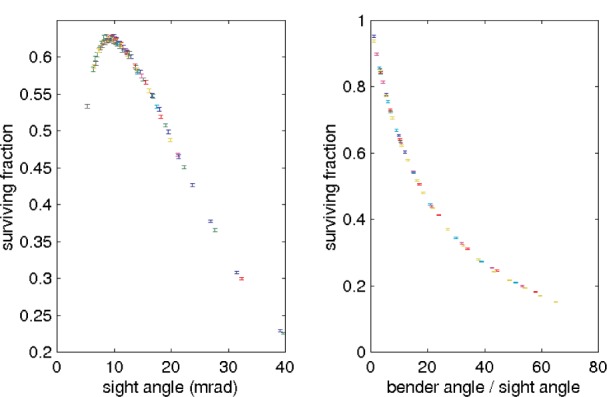
Universal behavior of benders. (a) Transmission through 100 mrad benders with different sight angles. The optimum sight angle is around 9.5 mrad. (b) Transmission through benders with the same sight angle but different lengths. Color in both cases represents benders of different radius; the radius was varied from 5 to 100 m. Uncertainties are statistical.

**Fig. 4:**
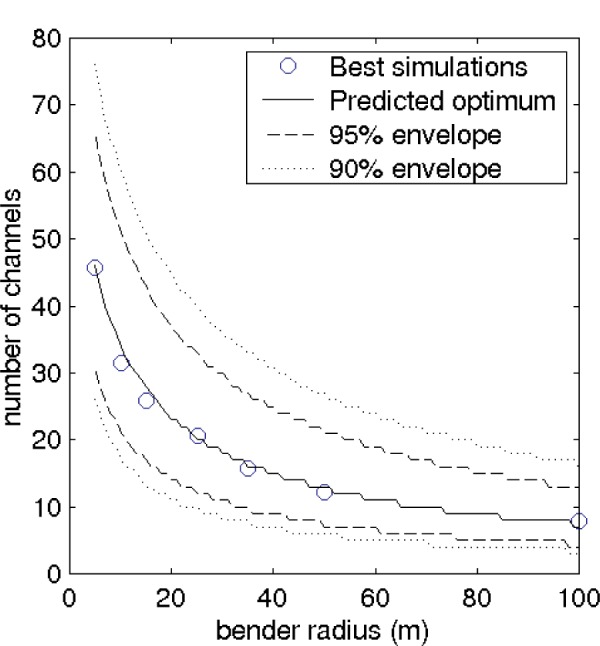
Optimal numbers of channels for benders of radius 5 – 100 m are shown by the solid curve. Benders are 4 cm wide and bend through 10 mrad. The dashed curves show the numbers of channels for which the exit flux is reduced by 5 % and 10 %. All the curves were obtained by combining the universal bender response in Fig. 3 with absorption on the vanes at the bender entrance; the circles indicate the best number of channels in a series of simulations at a given radius.

For a bender with a very large number of channels, loss on the vanes at the bender entrance becomes important. The universal transmission curve shown in Fig. 3, combined with this entrance loss, is an accurate predictor of the behavior of the guide. For example, a 70 m radius bender is found by this method to require 10 channels. The optimal number of channels as a function of radius is shown in Fig. 4.

### 3.4 Vertical Tapering

**Fig. 5:**
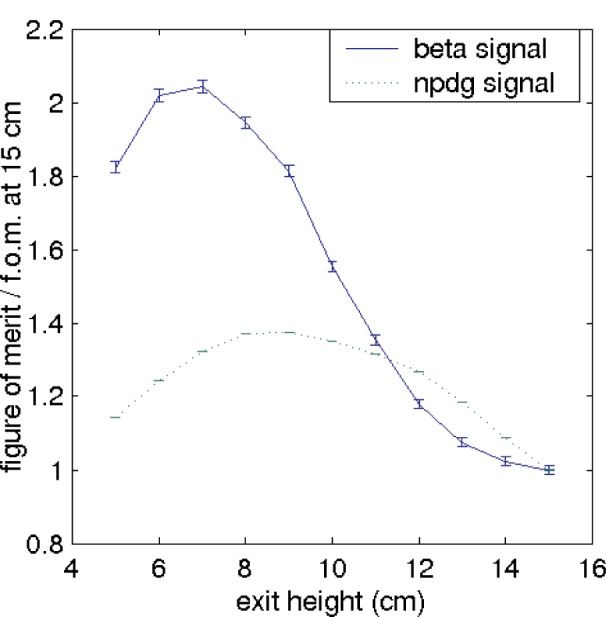
The effect of a simple vertical taper. See discussion in Sec. 1 for the definition of the figure of merit for the different experiments. Notice that the beta decay signal doubles as the vertical height of the beam is reduced by the taper to 7 cm. The shapes of the curves are independent of the bender radius. Uncertainties are statistical.

The CG4 guide was designed for a tall, narrow beam. However, both of the nuclear physics experiments described here require small cross-section neutron beams. Because our source is an *m* = 2 guide, we have the opportunity to use an *m* = 3 or *m* = 3.5 guide with a vertical taper to provide spatial compression. The response to this taper is shown in Fig. 5.

## 4. Conclusions

A curved, converging guide at the High Flux Isotope Reactor provides an attractive facility for the suggested experiments. The optimal guide configurations (with 1.1 m clearance) provided the following:

- 1 × 10^4^ n/cm^3^ in the abBA detector, and
- 3 × 10^10^ n/s in the NPDGamma detector
  (n.b. This is nearly an order of magnitude larger than the total fluence at LANSCE flight path 12).

These calculations deal only with the behavior of the guide and do not account for window losses etc. We note that modest additional gains may be expected by having a short bender followed by a straight tapered section. The use of a polygonal approximation to an elliptical focussing guide, rather than the simple taper investigated here, may improve performance as well.

## Figures and Tables

**Table 1:** Tradeoff between flux and floor space

Clearance (m)	Bender radius (m)	Guide transmission
0	∞	0.96
0.650	115.29	0.63
0.875	85.71	0.56
1.100	68.18	0.49
